# Retraction: *Camellia sinensis* methanolic leaves extract: Phytochemical analysis and anticancer activity against human liver cancer cells

**DOI:** 10.1371/journal.pone.0326075

**Published:** 2025-06-10

**Authors:** The PLOS One Editors

After this article [1] was published, concerns were raised about Figs 4 and 5. Specifically, multiple regions of similarities were noted within all panels of Fig 4 and within Fig 5(b). The corresponding author provided images stated to underlie Figs 4 and 5 as well as replacement image data for the panels of concern. However, assessment of these underlying and alternative images raised further image concerns.

During editorial reassessment of this article [1], PLOS identified additional concerns about misclassification of the HepG2 cell line reported in [1]. Specifically, this article [1] presents HepG2 as a ‘human hepatic cancer cell line’ and studies it in the context of hepatocellular carcinoma. However, prior to publication of this article [1], the HepG2 cell line was identified as a misclassified cell line, and was shown to be derived from a hepatoblastoma instead [2]. The corresponding author stated that they were not aware of the misclassification of the HepG2 cell line, and they did not provide material confirming the identity of the cell line used in this study. In the absence of a confirmed hepatocellular carcinoma cell line, the relevance of the results reported in [1] to hepatocellular carcinoma is in question.

Furthermore, a concern was also raised regarding data points which appear to fall outside the flow cytometry plot in Fig 3(a). The corresponding author stated these were likely due to a defect in printing but did not provide the original underlying data files and PLOS considers this concern unresolved.

In light of the unresolved concerns with Figs 4-5 that question the integrity and validity of the reported results and conclusions, the *PLOS One* Editors retract this article.

All authors did not agree with the retraction and stand by the article’s findings.
